# Immunotherapy – 2067. FEL d 1 peptide antigen desensitization safety and efficacy in a double-blind, placebo-controlled environmental exposure chamber study

**DOI:** 10.1186/1939-4551-6-S1-P150

**Published:** 2013-04-23

**Authors:** Roderick Peter Hafner, Piyush Patel, Annemarie Salapatek, Paul Laidler, Mark Larché, Deepen Patel

**Affiliations:** 1 Circassia Limited, Oxford, UK; 2 Allerpharma, Toronto, ON, Canada; 3 Cetero Research, Missisauga, ON, Canada; 4 Department of Medicine, Mcmaster University, Hamilton, ON, Canada

## Background

Allergic rhinoconjunctivitis is an increasing problem worldwide with significant impact on quality of life and productivity. Sensitivity to cats accounts for 10-15% of the disease burden. Previous immunotherapy studies with two 27aa peptides were unsuccessful as a result of early and late phase responses. Cat Peptide Antigen Desensitisation (Cat-PAD) is a mixture of seven T-cell epitopes (13-17aa) derived from Fel d1. This study evaluated safety and relationship between dose, dosing regimen and symptom scores in cat allergic subjects with rhinoconjunctivitis 17-21weeks(wk) after starting treatment using a standardized allergen challenge in an Environmental Exposure Chamber (EEC).

## Methods

In a multicentre, double-blind, placebo-controlled clinical trial, subjects attended an EEC, before and after treatment. 121 subjects were randomised to one of four treatment regimens (Cat-PAD: 4x3nmol 2wk apart, 4x6nmol 2wk apart, 4x3nmol 4wk apart, 8x3nmol 2wk apart) or placebo. Clinical efficacy was assessed by measurement of changes in Total Rhinoconjunctivitis Symptom Score (TRSS) during EEC visits. Safety was assessed by observing subjects in the clinic for 1 hour on each dosing day and capturing adverse events (AE) by direct questioning of subjects at every visit.

## Results

There were no Serious Adverse Events. Frequencies of all Treatment Emergent Adverse Events (TEAE) in the Cat-PAD treatment arms were less than in the Placebo cohort with the exception of the 6nmol cohort which trended slightly higher. Analysis of the respiratory system TEAEs showed no evidence of any safety signal after treatment with Cat-PAD. Respiratory system TEAEs, including asthma, dyspnoea and wheezing, occurred at a low frequency in both active and placebo groups, with no obvious difference between the groups. Treatment with Cat-PAD showed greater efficacy when dosed over 12-14wk than when dosed over 6wk. 8x3nmol dose showed a statistically significant reduction in symptoms vs placebo (p <0.05) in subjects who attended the main centre for all their visits. The 6nmol dose showed a trend to be superior to the 3nmol dose, albeit tested in a sub-optimal regimen.

## Conclusions

Cat-PAD was safe and well tolerated and improved TRSS. Potential for greater treatment benefits by using a higher dose over 12-14wk should be evaluated in future studies.

